# Development and Cytomolecular Identification of Monosomic Alien Addition and Substitution Lines of Triticale (×*Triticosecale* Wittmack) With 2S^k^ Chromosome Conferring Leaf Rust Resistance Derived From *Aegilops kotschyi* Boiss

**DOI:** 10.3389/fpls.2020.509481

**Published:** 2020-12-14

**Authors:** Michał T. Kwiatek, Waldemar Ulaszewski, Jolanta Belter, Dylan Phillips, Roksana Skowrońska, Aleksandra Noweiska, Halina Wiśniewska

**Affiliations:** ^1^Department of Genomics, Institute of Plant Genetics of the Polish Academy of Sciences, Poznañ, Poland; ^2^Institute of Biological, Environmental and Rural Sciences, Aberystwyth University, Aberystwyth, United Kingdom; ^3^Department of Genetics and Plant Breeding, Poznañ University of Life Sciences, Poznañ, Poland

**Keywords:** back-cross, fluorescence in situ hybridization, molecular markers, monosomic, resistance genes, triticale 2, Aegilops kotschyi

## Abstract

Alien chromosome introgression has become a valuable tool to broaden the genetic variability of crop plants via chromosome engineering. This study details the procedure to obtain monosomic addition and monosomic substitution lines of the triticale carrying 2S^k^ chromosome from *Aegilops kotchyi* Boiss., which harbors *Lr54* + *Yr37* leaf and stripe rust-resistant gene loci, respectively. Initially, *A. kotschyi* × *Secale cereale* artificial amphiploids (2*n* = 6*x* = 42 chromosomes, UUSSRR) were crossed with triticale cv. “Sekundo” (2*n* = 6*x* = 42, AABBRR) in order to obtain fertile offspring. Cyto-molecular analyses of five subsequent backcrossing generations revealed that 2S^k^ chromosome was preferentially transmitted. This allowed for the selection of monosomic 2S^k^ addition (MA2S^k^) lines of triticale. Finally, the 2S^k^(2R) substitution plants were obtained by crossing MA2S^k^ with the nullisomic (N2R) plants of triticale. The presence of 2S^k^ chromosome in subsequent generations of plants was evaluated using SSR markers linked to *Lr54* + *Yr37* loci. Disease evaluation of the monosomic 2S^k^(2R) substitution plants for the reaction to leaf and stripe rust infection were carried out under controlled conditions in a growth chamber. The results showed significant improvement of leaf rust resistance severity of monosomic substitution plants compared with control (“Sekundo”). In contrast, the introgression of the *Lr54* + *Yr37* loci did not lead to improvement of stripe rust resistance. In summary, the creation of monosomic addition and monosomic substitution lines of triticale is the starting point for the precise and guided transfer of *Lr54* + *Yr37* loci. The results showed that the developed materials could be exploited for the development of triticale varieties with resistance to leaf rust.

## Introduction

Wild relatives and related species with homoeologous genomes are important for broadening the genetic variability of crop plants. For many crops, wild relatives provide a vast reservoir for most of agronomically important traits. A large number of the highest yielding wheat (*Triticum aestivum* L.) cultivars carry portions of alien chromatin introgression from related crops (*Secale cereale* L.) or weedy species (*Aegilops* sp., *Dasypyrum* sp., *Elymus* sp. *Haynaldia* sp.) (Gill et al., 2011). The *Aegilops* genus is the closest wild relative of bread wheat (*T. aestivum*; 2*n* = 6*x* = 42 chromosomes; AABBDD) or triticale (× *Triticosecale* Wittmack; 2*n* = 6*x* = 42; AABBRR). It contains 11 diploid, 10 tetraploid, and 2 hexaploid species and provides a vast reservoir of valuable genes, which were eliminated during the domestication and breeding of cultivated cereals.

*Aegilops kotschyi* Boiss. (U^k^U^k^S^k^S^k^, 2*n* = 4*x* = 28) is valuable from genetic and breeding point of view as it carries genes associated with disease resistance, drought, heat, salt tolerance, as well as other beneficial traits (Schneider et al., 2008). Rawat et al. (2009) utilized *A. kotschyi* as a source of genes to increase the iron and zinc content in seeds of wheat. *A. kotschyi* was crossed as a male parent with bread wheat line “Chinese Spring” lacking the *Ph1* locus, which is a suppressor of homeologous pairing in wheat (Riley and Chapman, 1958). The aim of their study was to bring the genes associated with increased iron and zinc content in seeds, harbored in the 2S^k^ and 7U^k^ chromosomes of *A. kotschyi*, into the genome of wheat. They obtained only a 2S^k^ and 7U^k^ chromosome substitution line, which were not very suitable for commercial exploitation due to linkage drag. It is also reported that chromosome 2S^k^ of *A. kotschyi* possess leaf and stripe rust-resistant loci of *Lr54* and *Yr37* genes (Marais et al., 2005; Prażak and Paczos-Grzęda, 2013). Marais et al. (2005) have introduced *A. kotschyi*-derived leaf and stripe rust-resistant genes *Lr54* and *Yr37* to the wheat genome by the induction of 2DS.2S^k^L chromosome translocation.

Polyploids, such as of cultivated forms wheat or triticale, contain multiple sets of chromosomes and have highly buffered genotypes that are more permissive to benefit from alien introgression than diploids (Qi et al., 2007). Monosomic alien addition lines (MAALs) contain only one alien chromosome in addition to the receptor background chromosomes. The production of monosomic addition lines rely on backcrosses with an acceptor crop (Faris et al., 2002; Brar and Dhaliwal, 2004; Wulff and Moscou, 2014; Kwiatek and Nawracała, 2018). In traditional breeding of intraspecific hybrids, the recurrent backcrossing is commonly employed to transfer alleles at one or more loci from a donor to an elite variety (Veatch-Blohm, 2007). The expected recurrent parent genome recovery would be 99.2% at the end of six backcrossing, which is most similar to improved variety. The proportion of the recurrent parent genome is recovered at a rate of 1 − (1/2)*^t^* ^+^^1^ for each of the generations of backcrossing (Singh and Singh, 2015). When generating interspecific hybrids, backcrossing is required to generate addition, substitution, and translocation lines. In wheat and triticale, chromosome pairing between homologous chromosomes is strictly regulated by the expression of *Ph1* and *Ph2* loci (Riley and Chapman, 1958; Sears, 1977). Thus, subsequent backcrosses eliminate the alien chromatin gradually. However, the alien chromosome can be transmitted to the offspring preferentially, which contributes to the saturation of specific chromosomes (Endo, 2007).

Some *Aegilops* chromosomes were reported to be transmitted preferentially to the offspring in the process of backcrossing to produce alien chromosome addition lines of wheat (Endo, 2007) and triticale (Kwiatek et al., 2016c). The gametocidal activity of genes located on chromosome 4S^sh^ from *A. sharonensis* Eig is an example of when a chromosome is preferentially eliminated (Endo, 1990). Such chromosomes were reported to carry gametocidal factors (*Gc*s) (Endo, 1985). These chromosomes are known to remain in host plants in a “selfish” way. The preferential transmission of *Gc* chromosomes result from the elimination of gametes that lack them, while the gametes with the *Gc* chromosomes remain fertile (Nasuda et al., 1998). *Gc* factors cause extensive chromosome breakage, which results in the induction of non-functional gametes and exclusive transmission of the *Gc* chromosome to the offspring (Nasuda et al., 1998). *Gc*s have been reported in *A. geniculata*, *A. triuncialis*, *A. caudata*, *A. cylindrica, A*. *longissima*, *A. sharonensis*, and *A. speltoides*. Their activity is mostly confined to 2, 3, and 4 homeologous groups of C, S, S^1^, S^sh^, and M^g^ genomes. It has been reported that *Gc* genes can be constructively utilized for development of addition, deletion and translocation stocks in wheat (Endo, 2007) and triticale (Kwiatek et al., 2017b).

Monosomic alien addition and substitution lines (MAALs and MASLs) are widely used as linking bridges for the transfer of desirable genes from wild species into elite cultivars. For example, wheat-rye addition, substitution, and translocation lines are used as pre-breeding materials for the improvement of wheat (Lukaszewski, 2000, 2016) and triticale (Lukaszewski, 2006). MASLs can be used to identify favorable genes in wild species, allowing for more accurate and rapid transfer of such genes to create introgression lines. Such initiatives provide the opportunity to examine the effect of specific alien (Edet et al., 2018) and to construct the physical maps of specific chromosomes (Kynast et al., 2001). MASLs are produced by crossing monosomic or nullisomic acceptors with appropriate addition lines (Sears, 1972). The missing chromosome of acceptor plant is substituted by the donor homoeologue. The production of substitution lines for alien chromosomes is the initial step for direct introgression of specific alien chromosome segments into the acceptor genome (Lukaszewski, 2000, 2017). One of the most common procedures that reduce the amount of alien chromosome present is the induction of crop-alien Robertsonian translocations (RobTs). This approach requires a set of specific genetic stock plants, including MAALs or MASLs as a donor, and monosomic plants with a single acceptor chromosome (Kwiatek et al., 2017b).

The main aim of this study was to generate the monosomic addition and substitution line of triticale with introgression of alien 2S^k^ chromosome derived from *A. kotschyi*, which could be used for efficient study and transfer of genes responsible for leaf and stripe rust resistance.

## Materials and Methods

### Plant Material

**FIGURE 1:**
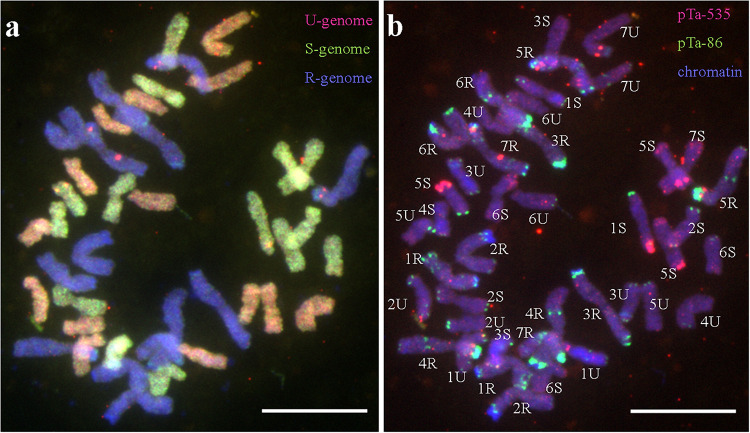
Flourescence/genomic *in situ* hybridization performed on mitotic chromosomes of *A. kotschyi* × *S. cereale* amphiploid. **(a)** U^k^- and S^k^-genome chromosomes are labeled with Atto-550 (red) and Atto-488 (green), respectively. R-genome chromosomes labeled with DAPI (blue). **(b)** Probes pTa-535 and pTa-86 are labeled with Atto-550 (red) and Atto-488 (green), respectively. Chromosomes counterstained with DAPI (blue). Scale bar, 10 μm.

**FIGURE 2:**
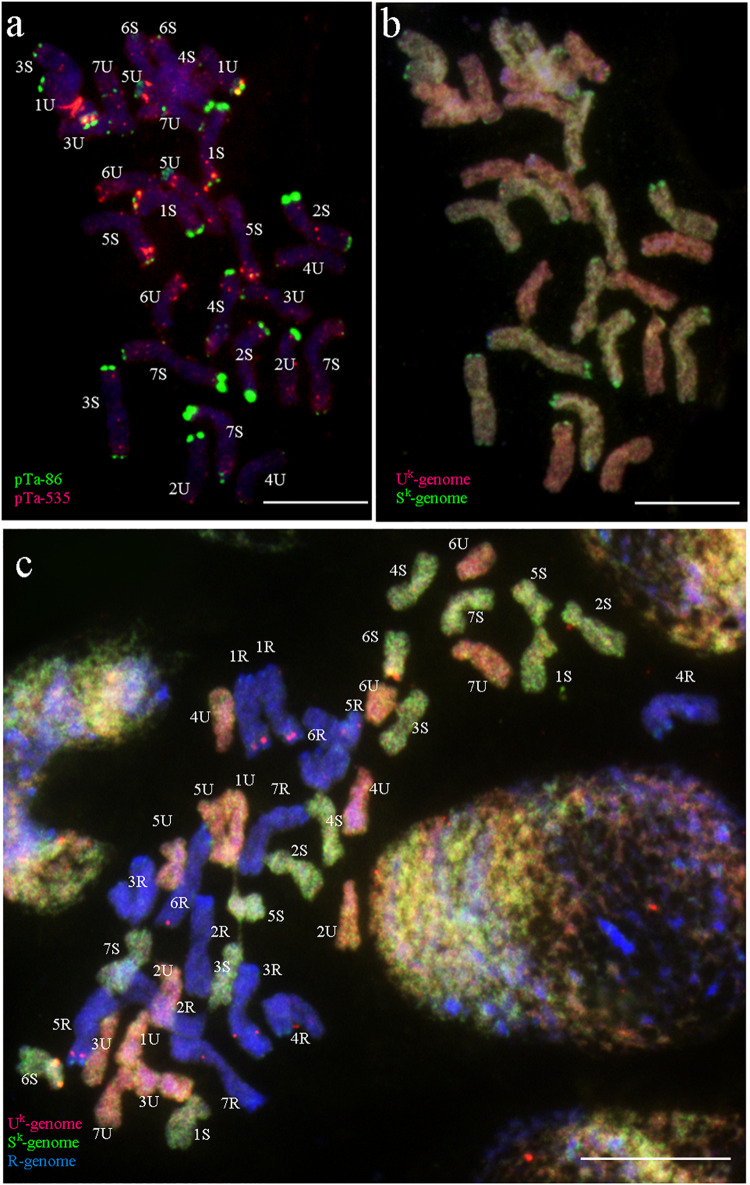
Fluorescence/genomic *in situ* hybridization performed on mitotic chromosomes of *A. kotschyi* Boiss (2*n* = 6*x* = 42; U^k^U^k^S^k^S^k^; no. 14808 using **(a)** pTa-86 (Atto-488, green), pTa-535 (Atto-550, red). Genomic *in situ* hybridization using U-genome (Atto-550, red) and S-genome (Atto-488, green) probes performed on mitotic chromosomes of **(b)** *A. kotschyi* and **(c)** *Aegilops kotschyi* × *Secale cereale* (2*n* = 6*x* = 42; UUMMRR). **(a,c)** Chromosomes counterstained with DAPI (blue). Scale bar, 10 μm.

**FIGURE 3:**
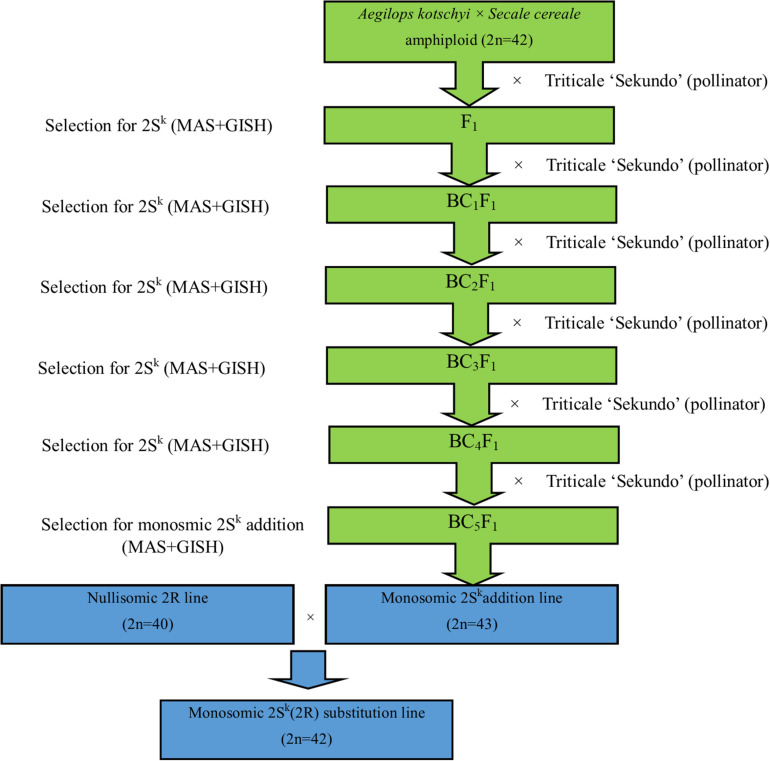
Pedigree of monosomic 2S^k^(2R) substitution plants of triticale “Sekundo.”

An artificial amphidiploid line of *Aegilops kotschyi* × *Secale cereale* (2*n* = 6*x* = 42; U^k^U^k^S^k^S^k^RR) (Figure 1) was obtained by Wojciechowska and Pudelska (2002) from a cross between *A. kotschyi* Boiss. (no. 14,808; kindly provided from Prof. Taihachi Kawahara, Kyoto University, Kyoto, Japan; Figures 2a,b), and diploid rye “Dankowskie Złote” (Danko Hodowla Roślin sp. z o.o.). Triticale “Sekundo” (Danko Hodowla Roślin Sp. z o.o.) was used as a pollinator for F_1_ seed production and five subsequent backcrosses (Figure 3). Nullisomic (N2R) line of triticale were developed by Kwiatek et al. (2016c) and used for crossing with monosomic alien addition of 2S^k^ chromosome to produce monosomic 2S^k^(2R) substitution plants of triticale (Figure 3). Cross-hybridization was made in greenhouse of the Institute of Plant Genetics of the Polish Academy of Sciences. Anthers of maternal plants were emasculated and spikes isolated using paper bags, in order to avoid uncontrolled pollination. Mature stigmas were pollinated with the pollen of triticale “Sekundo.” Crossing efficiency percentage was calculated by dividing of the total amount of seeds with total number of pollinated flowers.

### Cytogenetic Studies

Accumulation of cells at mitotic metaphase and fixation was carried out according to Kwiatek et al. (2017b). Root meristems were digested at 37°C for 2 h and 40 min in an enzymes solution containing 0.2% (*v*/*v*) Cellulase Onozuka R-10 and Calbiochem cytohelicase (1:1 ratio) and 20% pectinase (Sigma), in 10 mM citrate buffer (pH 4.6). Digested root tips were placed on slides with a drop of ice-cold 60% acetic acid and squashed. The coverslips were removed with a razor after liquid nitrogen treatment.

### Probe Preparation and Fluorescence *in situ* Hybridization

Total genomic DNA was purified using GeneMATRIX Plant & Fungi DNA Purification Kit (EURx, Gdańsk, Poland). DNA of *Aegilops sharonensis* Eig (a progenitor of the S^k^-genome of *A. kotschyi*; PI 551020, U.S. National Plant Germplasm System) and *A. umbellulata* (a progenitor of the U^k^-genome of *A. kotschyi*; PI 298905, U.S. National Plant Germplasm System) were labeled by nick translation with Atto-488NT and Atto-550NT kits (Jena Bioscience, Jena, Germany), respectively. Blocking DNA from triticale “Sekundo” was prepared by boiling for 30–45 min (1:50 probe:block ratio). Chromosomes were identified using fluorescence *in situ* hybridization (FISH) with the repetitive sequences from pTa-86, pTa-535, pTa-103 (centromere specific), and pTa-k374 (homologous to ribosomal DNA sequence 28S) clones described by Komuro et al. (2013). Clones were amplified from genomic DNA of “Chinese Spring” wheat (Kwiatek et al., 2016b; Goriewa-Duba et al., 2018) and labeled using Atto-488NT, Atto-550NT, and Atto-647NT nick translation kits (Jena Bioscience, Germany). FISH/genomic *in situ* hybridization (GISH) experiments were performed according to Kwiatek et al. (2017b). Slides were examined with the Olympus BX 61 automatic epifluorescence microscope equipped with Olympus XM10 CCD camera. Image processing was performed using Olympus Cell-F (version 3.1; Olympus Soft Imaging Solutions GmbH: Münster, Germany). Chromosomes of *Aegilops* and triticale were identified by comparing the signal patterns of the probes (Badaeva et al., 2004; Komuro et al., 2013; Kwiatek et al., 2013; Ruban and Badaeva, 2018).

### SSR Marker Analysis

The SSR marker S14-410 (forward primer: 5′ -ACCAATT CAACTTGCCAAGAG-3′; reverse primer: 5′-GAGTAACATG CAGAAAACGACA-3; Smit, 2013) closely linked to the *Lr54* + *Yr37* loci on the 2S^k^ chromosome was used to genotype genomic DNA of the plant materials. Genomic DNA of *Aegilops kotschyi*, triticale “Sekundo” and the hybrid plants were isolated using Plant DNA Purification Kit (EurX Ltd., Gdañsk, Poland). PCR reactions were performed in a LabCycler thermal cycler (SensoQuest Biomedizinische Elektronik, Goettingen, Germany). The PCR reaction consisted of 150 nM each primer (Merck KGaA, Darmstadt, Germany), 0.2 mM of each nucleotide, 1.5 mM MgCl_2_, 0.2 units of Taq-DNA hot-start polymerase (TaqNovaHS, Blirt, Gdańsk, Poland), and 50 ng of genomic DNA as a template. The PCR conditions were as follows: 5 min at 95°C, then 35 cycles of 30 s at 94°C, 30 s at 61°C, 1 min at 72°C, and 5 min at 72°C. Midori Green Direct (Nippon Genetics Europe) was added to each amplification product and analyzed by gel electrophoresis on 2% agarose gel (LabEmpire, Poznań, Poland). Gels were visualized and photographed using EZ GelDoc System (BioRad, Hercules, CA, United States).

### Evaluation of Leaf Rust Symptoms in Growth Chamber

**FIGURE 4:**
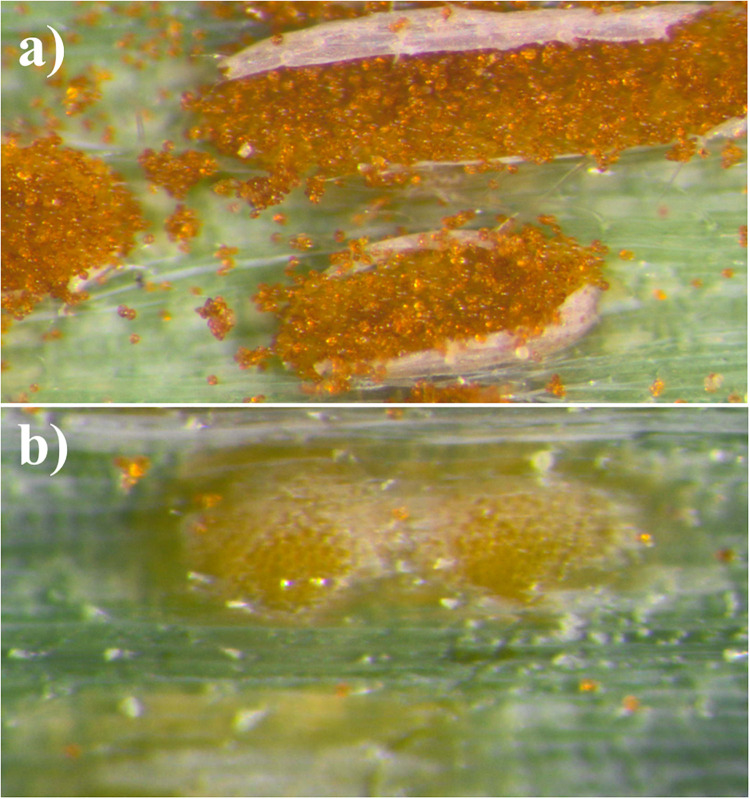
Symptoms of **(a)** leaf rust and **(b)** stripe rust observed on leaves of triticale “Sekundo.”

Evaluation of the response of monosomic substitution plants on leaf rust infection was conducted in the growth chamber (at IPG PAS) using leaf and stripe rust urediniospores, which were collected separately from triticale fields in three localizations in Wielkopolska region: IPG PAS Experimental Station in Cerekwica, Poland (52° 31° 41°41°04° 25° 54 Figure 4). Both experiments were repeated. Each experiment repeat included forty monosomic 2S^k^(2R) substitution plants and forty control plants (“Sekundo”), which were sprayed with leaf rust urediniospore solution containing 0.1% Tween 20, at three-leaf stage. Another 160 plants were sprayed with stripe rust urediniospore solution. The inoculated plants were then incubated in a humid growth chamber free from light for 10 days. After inoculation, the plants were maintained under a day/night photoperiod of 18/6 h, a temperature of 16–22°C. Winter triticale cv. “Sekundo” was taken as the susceptible control. The infection type of each individual was scored at three timepoints [10, 15, and 20 days post-inoculation (*dpi*)] using an infection scale adapted from Roelfs (1988) and transformed into nine-grade scale (1, high resistance; 9, susceptibility, Tables 2, 3; McNeal et al., 1971). The phenotypic data were analyzed using analysis of variance (ANOVA) and Tukey’s highest significant difference (HSD) test.

## Results

### Production of Monosomic 2S^k^ Addition Plants From Triticale-*A. kotschyi* Hybrids

**FIGURE 5:**
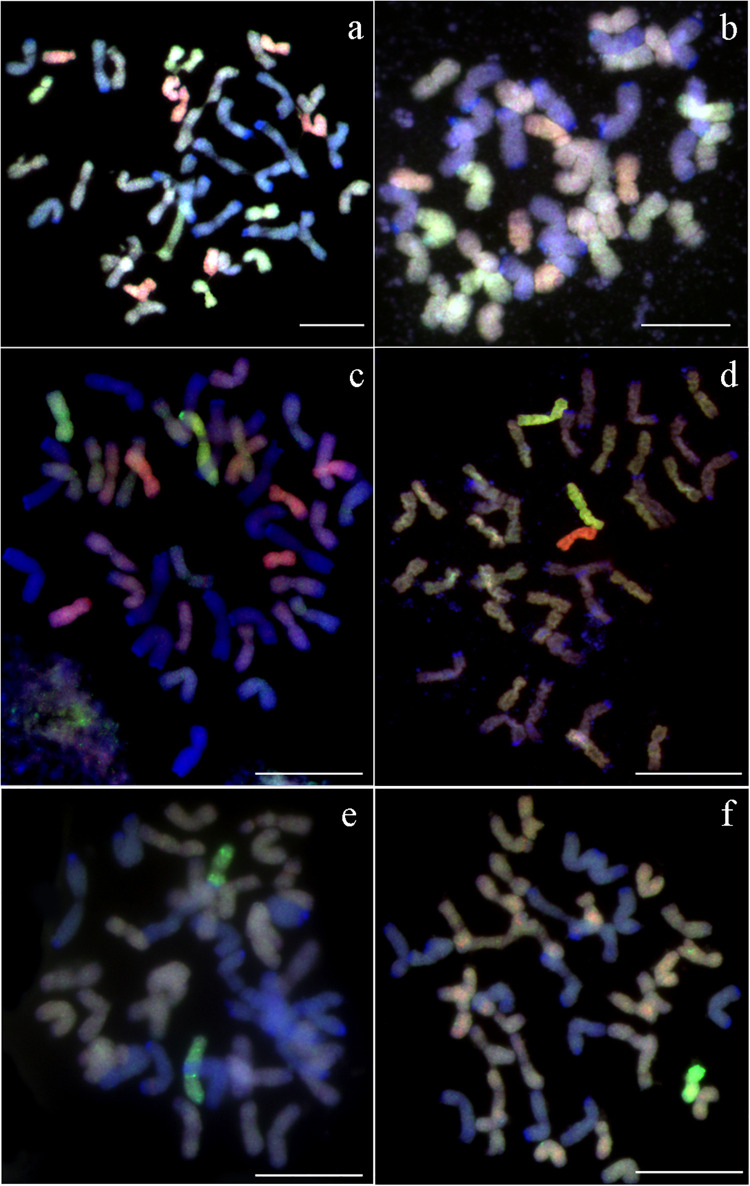
Genomic *in situ* hybridization performed on mitotic chromosomes of **(a)** F_1_, **(b)** BC_1_F_1_, **(c)** BC_2_F_1_, **(d)** BC_3_F_1_, **(e)** BC_4_F_1_, and **(f)** BC_5_F_1_ triticale-*Aegilops* introgression plants. A- and B-genome chromosomes are labeled with DAPI (light blue), as well as R-genome chromosomes (dark blue). U^k^- and S^k^-genome chromosomes are labeled with Atto-550 (red) and Atto-488 (green), respectively. Scale bar, 10 μm.

**FIGURE 6:**
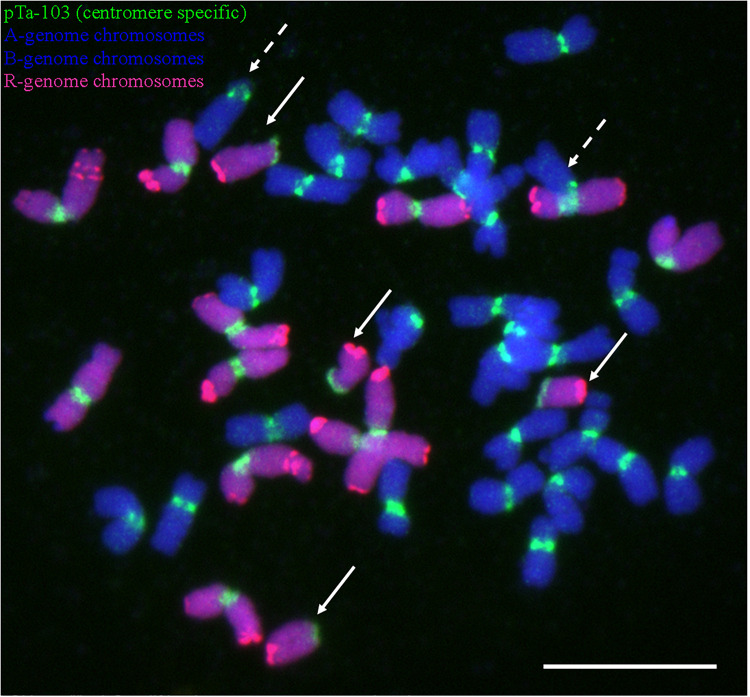
Centric breaks of chromosomes in BC_5_F_1_ plant screened using multi-color GISH/FISH. Centromere regions were mapped using pTa-103 (Atto-488, green) probe. Dotted arrows, chromosome aberrations of A- or B-genome (DAPI, blue) of triticale; solid arrows, chromosome aberrations of R-genome (Atto-550, red) of triticale. Scale bar, 10 μm.

Seven F_1_ plants were produced from a cross between hexaploid triticale “Sekundo” (2*n* = 6*x* = 42 chromosomes; AABBRR) and hexaploid *A. kotschyi* × *S. cereale* (2*n* = 6*x* = 42; UUSSRR; Figure 2c) hybrid, as a pollen donor. All seven F_1_ plants possessed 42 chromosomes including a complete haploid set of chromosomes of *A. kotschyi* (Figure 5a and Table 1). The number of R-genome chromosomes was 14. Fifteen BC_1_F_1_ seeds were obtained from 375 flowers of F_1_ plants, which were backcrossed using triticale pollen. FISH experiments revealed that chromosomes 1U^k^, 2U^k^, and 7S^k^ were eliminated (Figure 5b and Table 1). Fiftyone BC_2_F_1_ seeds were obtained by subsequent backcrossing of BC_1_F_1_ with triticale “Sekundo.” Fifty BC_2_F_1_ plants were karyotyped (Table 1), with the progressive elimination of *Aegilops* chromosomes was observed. The number of U-genome chromosomes varied between 1 and 2. The number of S^k^-genome chromosomes was much more diversified and varied between 2 and 6 (Figure 5c and Table 1). However, two chromosomes (2S^k^ and 4S^k^) were present in all 50 BC_2_F_1_ plants (Table 1). The BC_2_F_1_ plants were pollinated with triticale “Sekundo” pollen, and a total number of 234 seeds were obtained. FISH was carried out on 100 BC_3_F_1_, revealing that chromosome 7U^k^ was present in all plants. Chromosome number of S^k^-genome varied between 2 and 3 (Figure 5d). As before, chromosomes 2S^k^ and 4S^k^ were present in all 100 BC_3_F_1_ plants (Table 1). Fifty randomly selected plants of 93 BC_4_F_1_ hybrids were karyotyped using FISH and GISH techniques. Chromosomes of U^k^-genome were completely eliminated, but surprisingly chromosomes 2S^k^ and 4S^k^ were present in all 50 BC_4_F_1_ plants (Figure 5e and Table 1). The last backcross resulted in formation of 216 BC_5_F_1_ seeds (Table 1). One hundred plants were karyotyped and 99 of them carried additional 2S^k^ chromosomes (2*n* = 43) (Figure 5f and Table 1). Chromosome 2S^k^ was not present in one BC_5_F_1_ plant (2*n* = 42) (Table 1), and this plant showed centric breaks in three pairs chromosomes (Figure 6). Therefore, an investigation of six generations of triticale Sekundo × (*A. kotschyi* × *S. cereale*) hybrids (F_1_ to BC_5_F_1_) by means of molecular cytogenetic methods (FISH and GISH) revealed subsequent elimination of *Aegilops* chromosomes.

**TABLE 1:** Frequencies of individual *Aegilops kotschyi* chromosomes in five subsequent generations of backcrossing with triticale.

Generation	Pedegree	Number of pollinated flowers	Number of seeds	Number of plants (FISH)	Number of plants with													

					U-genome chromosomes							S-genome chromosomes						
		
					1	2	3	4	5	6	7	1	2	3	4	5	6	7
F_1_	Sekundo × AkSc*	710	7	7	7	7	7	7	7	7	7	7	7	7	7	7	7	7
BC_1_F_1_	F1 × Sekundo	375	15	15	0	0	13	2	11	13	15	1	15	12	15	5	1	0
BC_2_F_1_	BC_1_F_1_ × Sekundo	488	51	50	0	0	0	42	0	0	50	5	50	41	50	23	36	0
BC_3_F_1_	BC_2_F_1_ × Sekundo	715	234	100	0	0	0	0	0	0	100	0	100	0	100	0	23	0
BC_4_F_1_	BC_3_F_1_ × Sekundo	480	94	50	0	0	0	0	0	0	0	0	50	0	50	0	0	0
BC_5_F_1_	BC4F_1_ × Sekundo	487	216	100	0	0	0	0	0	0	0	0	99	0	0	0	0	0

*AkSc, Aegilops kotschyi × Secale cereale amphiploid (2n = 6x = 42 chromosomes, U^k^U^k^S^k^S^k^RR).

**TABLE 2:** Means of leaf rust infection levels scored 10, 15, and 20 days post-inoculation (dpi).

Plant material	Experiment	No of plants tested	Means of infection levels			
			
			10 dpi	15 dpi	20 dpi	Mean ± SD
Monosomic 2S^k^(2R) substitution plants	1	40	2.60	3.00	3.30	2.97 ± 0.48
Triticale cv. “Sekundo”	1	40	5.90	6.43	6.88	6.40 ± 0.73
Monosomic 2S^k^(2R) substitution plants	2	40	2.70	2.98	3.20	2.96 ± 0.42
Triticale cv. “Sekundo”	2	40	5.86	6.45	6.95	6.43 ± 0.75

**F ratio = 1,267.62 (Supplementary Table 1).**

**TABLE 3:** Means of stripe rust infection levels scored 10, 15, and 20 days post-inoculation (dpi).

Plant material	Experiment	No of plants tested	Means of infection levels			
			
			10 dpi	15 dpi	20 dpi	Mean ± SD
Monosomic 2S^k^(2R) substitution plants	1	40	2.85	3.18	3.85	3.29 ± 0.77
Triticale cv. “Sekundo”	1	40	2.73	3.20	3.80	3.24 ± 0.79
Monosomic 2S^k^(2R) substitution plants	2	40	2.70	3.15	3.78	3.21 ± 0.79
Triticale cv. “Sekundo”	2	40	2.67	3.30	4.00	3.33 ± 0.85

*F ratio = 0.5 (Supplementary Table 2).*

### Production of Monosomic 2S^k^(2R) Substitution Plants From Triticale-*A. kotschyi* Hybrids

**FIGURE 7:**
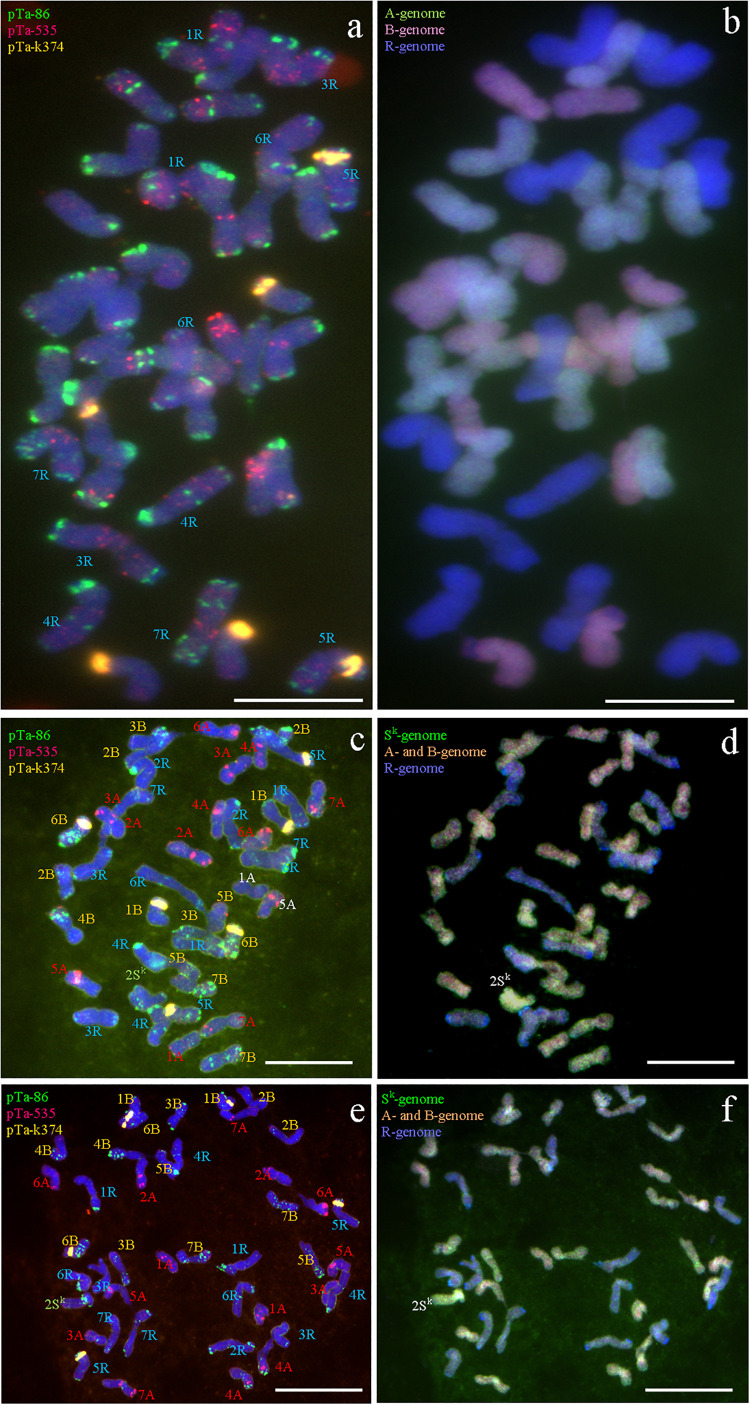
Fluorescence and genomic *in situ* hybridization performed on mitotic chromosomes of: **(a,b)** nullisomic 2R plant of triticale (2*n* = 40); **(c,d)** monosomic 2S^k^ addition plant of triticale (2*n* = 43); **(e,f)** monosomic 2S^k^(2R) substitution plant of triticale (2*n* = 42). pTa-86 (Atto-488; green), pTa-535 (Atto-550; red), and pTa-k374 (Atto-647; yellow) probes were used for FISH. Genomic probes of A-, B-, R-, and S^k^-genome were used for GISH. Scale bar, 10 μm.

A total number of 468 seeds were obtained from a cross between nullisomic N2R triticale plants (2*n* = 40; Figures 7a,b) and monosomic 2S^k^ addition line (MA2S^k^AL) of triticale (Figures 7c,d), as a pollen donor. 6,486 flowers of N2R plants were emasculated and pollinated with MA2S^k^ pollen. The crossing efficiency was 7.22%, and 468 plants were obtained. The FISH/GISH analyses revealed that each offspring plant carried 42 chromosomes, including 2R and 2S^k^ chromosomes, which were in monosomic condition (Figure 7e,f). The presence of monosomic or nullisomic 2R chromosome was not observed.

### Lr54 + Yr37 SSR Markers Analysis

**FIGURE 8:**
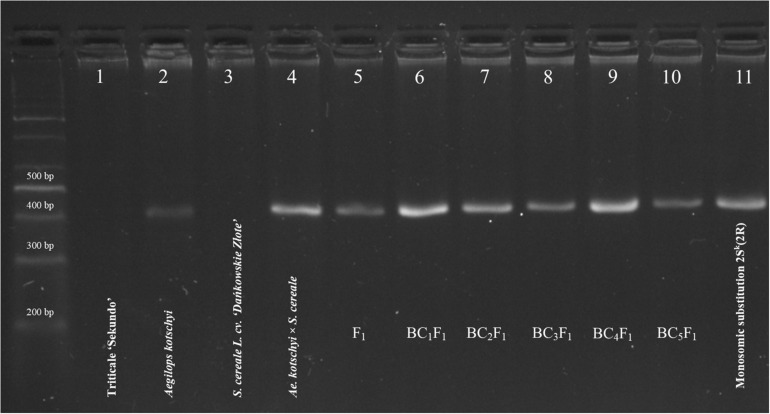
Amplification products (410 bp) of S14-410 marker linked to loci of *Lr54* + *Lr37* leaf and stem rust resistance genes on 2S^k^ chromosome of *Ae. kotschyi*. (1) triticale “Sekundo” (AABBRR); (2) *Aegilops kotschyi* (U^k^U^k^S^k^S^k^); (3) *Secale cereale* L. “Dañkowskie Złote”; (4) *Aegilops kotschyi* × *Secale cereale* (2*n* = 6*x* = 42; UUMMRR); (5) F_1_; (6) BC_1_F_1_; (7) BC_2_F_1_; (8) BC_3_F_1_; (9) BC_4_F_1_; (10) BC_5_F_1_; and (11) monosomic 2S^k^(2R) substitution plant of triticale (2*n* = 42). Size standard: Thermo Scientific^TM^ GeneRuler^TM^ 100 bp DNA Ladder.

PCR with SSR marker S14-410, linked to *Lr54* + *Yr37* loci, was conducted on *A. kotschyi*, triticale “Sekundo,” the monosomic substitution M2S^k^ (M2R) line and nullisomic N2R triticale plants (2*n* = 40). The same protocol was used to examine the offspring derived by subsequent backcrossing. A 410-bp amplicon for the S14-410 marker was identified in the control sample of *A. kotschyi* and in all hybrid plants of F_1_ to BC_4_F_1_ generations (Figure 8). Screening for the presence of the S14-410 marker in 100 BC_5_F_1_ plants showed that 410 bp amplicon was present in 99 plants. Similar screening was carried out in 468 plants, created by cross-hybridization of N2R triticale plants (2*n* = 40) and M2S^k^AL plants (2*n* = 43). PCR reaction with the genomic DNA of all recombinant plants yielded a 410-bp fragment.

### Evaluation of Leaf Rust Symptoms

The leaf and stripe rust response of two sets of 80 plants of triticale monosomic 2S^k^(2R) substitution line was evaluated at the seedling stage in the growth chamber and compared with phenotypes of acceptor cultivar “Sekundo” as controls (Table 2). The mean scores for two replications of control plants (6.40 ± 0.73 and 6.43 ± 0.75) showed that the inoculation solution was effective for induction of the leaf rust infection. The mean score of three independent evaluations of infection level (10, 15, and 20 dpi) of two replications of monosomic 2S^k^(2R) substitution plants was similar (2.96 ± 0.42 and 2.97 ± 0.48) (Table 2). The differences were significant at α = 0.01 (Supplementary Table 1). The scores for two replications of the leaf rust response experiment did not differ significantly (Supplementary Table 1). In contrast, the stripe rust responses of the monosomic 2S^k^(2R) substitution plants and the control triticale cv. “Sekundo” at the three successive points did not differ significantly (α = 0.01) (Table 3). Plants of cv. “Sekundo” showed moderate stripe rust resistance (3.24 ± 0.79 and 3.33 ± 0.85), as well as monosomic substitution plants (3.21 ± 0.79 and 3.29 ± 0.77). ANOVA test for all thee timepoints showed that there is no statistically significant differences between stripe rust response of monosomic substitution plants compared with triticale “Sekundo” controls (Supplementary Table 2).

## Discussion

Alien and cultivated species are closely related and possess homeologous genomes (or sub-genomes). Hence, it is possible to introduce the desirable variation into crop plants using a backcrossing program. However, isolation barriers exist that prevent the formation of interspecific hybridization, and present a significant obstacle for the exploration of alien genetic variation in crop improvement. The generation of artificial amphidiploids can facilitate the transmission of desirable genetic material from wild species to crops. In this study, synthetic *A. kotschyi* × *S. cereale* (2*n* = 6*x* = 42, U^k^U^k^S^k^S^k^RR) amdiphiploid plants were used as a donor of wild genetic material to widen the genetic variation in hexaploid triticale “Sekundo” (2*n* = 6*x* = 42, AABBRR).

Similar artificial amphidiploid forms were used to transfer *Lr32* and *Lr39* leaf rust resistance genes from *A. tauschii* × *S. cereale* (2*n* = 4*x* = 28; DDRR) (Kwiatek et al., 2015) and *Pm13* powdery mildew resistance gene from *A. variabilis* × *S. cereale* (2*n* = 6*x* = 42, U^v^U^v^S^v^S^v^RR) (Kwiatek et al., 2016a) into cultivated triticale. It was reported that the presence of an R-genome chromosome set resulted in semi-fertile F_1_ plants that were capable of producing an F_1_ via cross-hybridization of artificial *Aegilops biuncialis* × *S. cereale* (2*n* = 6*x* = 42, U^b^U^b^M^b^M^b^RR) amphiploid and hexaploid triticale (Kwiatek et al., 2017a). In this study, seven F_1_ plants were obtained via cross-hybridization of *A. kotschyi* × *S. cereale* and triticale. The first backcross with triticale “Sekundo” resulted in rapid elimination of the *Aegilops* chromosomes. The elimination range of the U^k^-genome chromosomes was far higher when compared with the rate of S^k^-genome chromosome loss (Table 1). The elimination of alien chromosomes was also observed in the BC_2_F_1_ generation. Interestingly, only 7U^k^, 4U^k^, 2S^k^, and 4S^k^ chromosomes were present in all of BC_2_F_1_ plants. In subsequent generations of triticale hybrids, obtained via successive backcrosses, the number of *Aegilops* chromosomes was highly reduced and the plants of BC_4_F_1_ carried 2S^k^ and 4S^k^ chromosomes only.

This pattern could be a result of gametocidal action of the *Gc* locus on 2S^sh^ of *Aegilops sharonensis* (Endo, 1985). Moreover, similar “breaker” element described in *A. sharonensis* (Maan, 1975). These loci have been mapped to a region proximal to a block of sub-telomeric heterochromatin on chromosome 4S^sh^L (Knight et al., 2015). The diploid *A. sharonensis* (2*n* = 2*x* = 14; S^sh^S^sh^) is a direct ancestor and a donor of S^k^-genome of *A. kotschyi* (Kihara, 1954; Badaeva et al., 2004; Ruban and Badaeva, 2018). Investigations into the origin of *A. kotschyi* showed that the U^k^ and S^k^ subgenomes are very similar to the diploid progenitors: *A. umbellulata* (2*n* = 2*x* = 14; UU) and *A. sharonensis*, respectively (Zohary and Feldman, 1962; Zohary, 1999; Ozkan et al., 2001; Feldman and Levy, 2012). It is a distinct possibility that there are gametocidal loci on 2S^k^ and 4S^k^ chromosomes of *A. kotschyi*, and those loci could be orthologous to *A. sharonensis* analogs.

In the presented study, 99% of BC_5_F_1_ monosomic alien addition plants of triticale carried an additional 2S^k^ chromosome. Endo (2007) suggested, that when the gametocidal action is intense, gametes without the alien chromosome may suffer severe chromosome damages, resulting in sterility. In this study, one BC_5_F_1_ plant without 2S^k^ chromosomes suffered minor chromosome aberrations (Figure 3). It could be hypothesized that gametes without 2S^k^ can be fertile and develop into plants with chromosome aberrations as no chromosome fragment has been lost. This genetic phenomenon is of particular interest to breeders. Linking *Gc* loci with *Lr54* + *Yr37* leaf and stripe rust-resistant genes in triticale varieties would ensure maintenance of these traits in subsequent generations without the need for selection. But, on the other hand, triticale plants with centric chromosome breaks can be selected and used for the induction of Robertsonian translocations (RobTs) (Kwiatek and Nawracała, 2018).

The molecular analyses with the S14-410 marker confirmed that the preferential transmission of the 2S^k^ chromosome occurred in all hybrid plants, from the F_1_ generation to BC_4_F_1_. The *Lr54* + *Yr17* marker identification reflected the results of cytogenetic analysis of BC_5_F_1_ plants. The key result of this study was the development of monosomic 2S^k^(2R) substitution lines, which can be used for induction of chromosome translocations. There are several reports describing the methodology used for the production of monosomic substitutions. According to Faris et al. (2002), this can be achieved by crossing an alien chromosome addition line containing the gene of interest with a line that is monosomic for a homoeologous wheat chromosome. This approach was applied for development of 1RS.1DL chromosome translocation in triticale “Rhino” (Lukaszewski and Curtis, 2006). The 1RS.1DL translocation was later used for the induction of multi-breakpoint translocation lines (Lukaszewski, 2006, 2017).

It is reported that triticale is infected by the races specific to both: wheat and rye; however, it was noticed that triticale is more easily attacked by the wheat physiological forms of the rusts than by the rye ones (Arseniuk, 1996). After inoculation, a significant improvement of the resistance level was observed in introgressed plants in comparison with triticale cv. Sekundo plants. The mean level of leaf rust resistance was high for two independent replications of the experiment (2.96 ± 0.42 and 2.97 ± 0.48). Such low infection rate can be considered as a result of *Lr54* gene expression. In a similar study, Marais et al. (2005) developed a 2DS.2S^k^L wheat-*A. kotschyi* line (called S14 translocation). The authors reported 96% resistant plants (72 tested) which were tested for resistance to eight *Pt* pathotypes (UVPrt2, UVPrt3, UVPrt4, UVPrt5, UVPrt8, UVPrt9, UVPrt10 and UVPrt13) and two *Pst* pathotypes (6E16A- and 6E22A-) endemic to South Africa. Moreover, it was shown that the S14 translocation evidently had preferential transmission (Marais et al., 2005). Intriguingly, monosomic 2S^k^(2R) substitution triticale plants, as well as control plants revealed the same, moderate resistance against stripe rust. In general, it is reported that triticale is more resistant against stripe rust as wheat. Moreover, triticale infection by *P. stiiformis* is highly dependent on plant growth stage and pathogen race (Rodriguez-Algaba et al., 2020). Hence, it could be possible that triticale “Sekundo” possesses a partial resistance against stripe rust and *Yr37* did not increase it.

In this study, the monosomic 2S^k^(2R) substitution plants were obtained through crossing between monosomic 2S^k^ addition line of triticale (M2S^k^AL), containing *Lr54* loci, with another triticale line that was nullisomic for 2R chromosome pair (N2R). The cross-hybridization between M2S^k^AL and N2R plants resulted in no offspring with the 2R chromosome in a monosomic condition (M2R). Hence, it can be hypothesized that gametocidal action of 2S^k^ chromosome killed gametes lacking it. Moreover, it could be possible that the inhibiting or suppressing factor of the 2S^k^ gametocidal action in triticale is located on the 2R chromosome. It is already known that some cultivars of common wheat possess genes that suppress the function of the *Gc* factors. Tsujimoto and Tsunewaki (1985) reported that that a suppressor gene (*Igc1*), that controls the suppression of *Gc* gene action on chromosome 3C of *A. truncialis*, is located on 3B chromosome of wheat “Norin 26.” Similarly, Endo (1988) postulated that gametocidal action of the 4S chromosome of *A. sharonensis* or *A. longissima* is suppressed by the presence of 4B chromosome of wheat. It could be plausible that both the *Gc* gene and the suppressor loci are located on chromosomes of the same homoeologous group; however, these hypotheses require future investigation.

The classical methods basing on backcrossing and selection are still significant in plant breeding. There are several countries, which are considered as key crop producers (for example, France, Germany, Poland, etc.), place a strict set of regulations on the cultivation of genetically modified (GM) crops. Hence, classical breeding methods, enriched by the molecular (and cyto-molecular)-assisted selection and chromosome manipulation are still worthy endeavors. In addition, the genetic mechanisms that regulate the process of segregation distortion should be seriously taken into consideration as a natural tool for chromosome manipulation and engineering.

## Data Availability Statement

The raw data supporting the conclusions of this article will be made available by the authors, without undue reservation, to any qualified researcher.

## Author Contributions

MK: conceptualization, methodology, formal analysis, writing original draft preparation, project administration, and funding acquisition. MK, WU, JB, and HW: validation. MK, JB, WU, RS, and AN: investigation. MK and HW: resources and supervision. MK and WU: data curation. MK and DP: writing—review and editing. All authors contributed to the article and approved the submitted version.

## Conflict of Interest

The authors declare that the research was conducted in the absence of any commercial or financial relationships that could be construed as a potential conflict of interest.

## References

[B1] Arseniuk E. (1996). “Triticale diseases - a review,” in *Triticale: today and tomorrow*, eds Carnide V. P. Guedes-Pinto H. Darvey N. (Netherlands: Springer), 499–525. 10.1007/978-94-009-0329-6_65

[B2] Badaeva E. D. Amosova A. V. Samatadze T. E. Zoshchuk S. A. Shostak N. G. Chikida N. N. (2004). Genome differentiation in *Aegilops*. 4. Evolution of the U-genome cluster. *Plant Syst. Evol.* 246 45–76. 10.1007/s00606-003-0072-4

[B3] Brar D. S. Dhaliwal H. S. (2004). ““Chromosome manipulations for crop improvement.”,” in *Plant Breeding: Mendelian to Molecular Approaches*, eds Jain H. K. Kharkwal M. C. (Dordrecht: Springer Netherlands), 65–96. 10.1007/978-94-007-1040-5_4

[B4] Edet O. U. Kim J.-S. Okamoto M. Hanada K. Takeda T. Kishii M. (2018). Efficient anchoring of alien chromosome segments introgressed into bread wheat by new *Leymus racemosus* genome-based markers. *BMC Genet.* 19:18. 10.1186/s12863-018-0603-1 29587653 PMC5872505

[B5] Endo T. R. (1985). Two types of gametocidal chromosome of *Aegilops sharonensis* and *Ae. longissima*. *Jap. J. Genet.* 60 125–135. 10.1266/jjg.60.125 25217460

[B6] Endo T. R. (1990). Gc chromosomes and their induction of chromosome mutations in wheat. *Jpn. J. Genet.* 65, 135–152.

[B7] Endo T. R. (1988). Induction of chromosomal structural changes by a chromosome *Aegilops cylindrica* L. in common wheat. *J. Hered.* 79, 366–370. 10.1093/oxfordjournals.jhered.a110529 31581766

[B8] Endo T. R. (2007). The gametocidal chromosome as a tool for chromosome manipulation in wheat. *Chromosom. Res.* 15 67–75. 10.1007/s10577-006-1100-3 17295127

[B9] Faris J. D. Friebe B. Gill B. S. (2002). Wheat Genomics: Exploring the Polyploid Model. *Curr. Genomics* 3 577–591. 10.2174/1389202023350219

[B10] Feldman M. Levy A. A. (2012). Genome evolution due to allopolyploidization in wheat. *Genetics* 192 763–774. 10.1534/genetics.112.146316 23135324 PMC3522158

[B11] Gill B. S. Friebe B. R. White F. F. (2011). Alien introgressions represent a rich source of genes for crop improvement. *PNAS* 108 7657–7658. 10.1073/pnas.1104845108 21527718 PMC3093505

[B12] Goriewa-Duba K. Duba A. Kwiatek M. Wiśniewska H. Wachowska U. Wiwart M. (2018). Chromosomal distribution of pTa-535, pTa-86, pTa-713, 35S rDNA repetitive sequences in interspecific hexaploid hybrids of common wheat (*Triticum aestivum* L.) and spelt (*Triticum spelta* L.). *PLoS One* 13:192862. 10.1371/journal.pone.0192862 29447228 PMC5813972

[B13] Kihara H. (1954). Considerations on the evolution and distribution of *Aegilops Species* based on the analyser-method. *Cytologia* 19 336–357. 10.1508/cytologia.19.336

[B14] Knight E. Binnie A. Draeger T. Moscou M. Rey M.-D. Sucher J. (2015). Mapping the ‘breaker’ element of the gametocidal locus proximal to a block of sub-telomeric heterochromatin on the long arm of chromosome 4Ssh of Aegilops sharonensis. *Theor. Appl. Genet.* 128 1049–1059. 10.1007/s00122-015-2489-x 25748115 PMC4435904

[B15] Komuro S. Endo R. Shikata K. Kato A. (2013). Genomic and chromosomal distribution patterns of various repeated DNA sequences in wheat revealed by a fluorescence *in situ* hybridization procedure. *Genome* 56 131–137. 10.1139/gen-2013-0003 23659696

[B16] Kwiatek M. T. Nawracała J. (2018). Chromosome manipulations for progress of triticale (*×Triticosecale*) breeding. *Plant Breed.* 137 823–831. 10.1111/pbr.12652

[B17] Kwiatek M. T. Majka J. Majka M. Belter J. Wisniewska H. (2017a). Adaptation of the pivotal-differential genome pattern for the induction of intergenomic chromosome recombination in hybrids of synthetic amphidiploids within Triticeae tribe. *Front. Plant Sci.* 8:1300. 10.3389/fpls.2017.01300 28791037 PMC5524833

[B18] Kwiatek M. T. Wiśniewska H. Ślusarkiewicz-Jarzina A. Majka J. Majka M. Belter J. (2017b). Gametocidal factor transferred from *Aegilops geniculata* Roth can be adapted for large-scale chromosome manipulations in cereals. *Front. Plant Sci.* 8:409. 10.3389/fpls.2017.00409 28396677 PMC5366343

[B19] Kwiatek M. Belter J. Majka M. Wiśniewska H. (2016a). Allocation of the S-genome chromosomes of *Aegilops variabilis* Eig. carrying powdery mildew resistance in triticale (× *Triticosecale* Wittmack). *Protoplasma* 253 329–343. 10.1007/s00709-015-0813-6 25868512 PMC4783449

[B20] Kwiatek M. Majka M. Majka J. Belter J. Suchowilska E. Wachowska U. (2016b). Intraspecific polymorphisms of cytogenetic markers mapped on chromosomes of *Triticum polonicum* L. *PLoS One* 11:158883. 10.1371/journal.pone.0158883 27391447 PMC4938433

[B21] Kwiatek M. Majka M. Ślusarkiewicz-Jarzina A. Ponitka A. Pudelska H. Belter J. (2016c). Transmission of the *Aegilops ovata* chromosomes carrying gametocidal factors in hexaploid triticale (*×Triticosecale* Wittm.) hybrids. *J. Appl. Genet.* 57 305–315. 10.1007/s13353-015-0332-3 26825077 PMC4963450

[B22] Kwiatek M. Majka M. Wiśniewska H. Apolinarska B. Belter J. (2015). Effective transfer of chromosomes carrying leaf rust resistance genes from *Aegilops tauschii* Coss. into hexaploid triticale (*X Triticosecale* Witt.) using *Ae. tauschii × Secale cereale* amphiploid forms. *J. Appl. Genet.* 56 163–168. 10.1007/s13353-014-0264-3 25502891 PMC4412281

[B23] Kwiatek M. Wiśniewska H. Apolinarska B. (2013). Cytogenetic analysis of *Aegilops* chromosomes, potentially usable in triticale (*X Triticosecale* Witt.) breeding. *J. Appl. Genet.* 54 147–155. 10.1007/s13353-013-0133-5 23378244 PMC3620446

[B24] Kynast R. G. Riera-Lizarazu O. Vales M. I. Okagaki R. J. Maquieira S. B. Chen G. (2001). A complete set of maize individual chromosome additions to the oat genome. *Plant Physiol.* 125 1216–1227. 10.1104/pp.125.3.1216 11244103 PMC65602

[B25] Lukaszewski A. J. (2000). Manipulation of the 1RS.1BL translocation in wheat by induced homoeologous recombination. *Crop Sci.* 40 216–225. 10.2135/cropsci2000.401216x

[B26] Lukaszewski A. J. (2006). Cytogenetically engineered rye chromosomes 1R to improve bread-making quality of hexaploid triticale. *Crop Sci.* 46 2183–2194. 10.2135/cropsci2006.03.0135

[B27] Lukaszewski A. J. (2016). “Manipulation of homologous and homoeologous chromosome recombination in wheat,” in *Plant Cytogenetics: Methods and Protocols*, eds Kianian S. F. Kianian P. M. A. (New York, NY: Springer New York), 77–89. 10.1007/978-1-4939-3622-9_7 27511168

[B28] Lukaszewski A. J. (2017). A set of new 1RS translocations from wheat cv. Amigo in a uniform genetic background. *Euphytica* 213:214 10.1007/s10681-017-2008-z

[B29] Lukaszewski A. Curtis C. (2006). Transfer of the Glu−D1 gene from chromosome 1D of bread wheat to chromosome 1R in hexaploid triticale. *Plant Breed.* 109 203–210. 10.1111/j.1439-0523.1992.tb00174.x

[B30] Maan S. S. (1975). Exclusive preferential transmission of an alien chromosome in common wheat 1. *Crop Sci.* 15 287–292. 10.2135/cropsci1975.0011183X001500030002x

[B31] Marais G. F. McCallum B. Marais A. S. (2005). Leaf rust and stripe rust resistance genes *Lr54* and *Yr37* transferred to wheat from *Aegilops kotschyi*. *Plant Breed.* 124 538–541. 10.1007/s10681-006-9092-9

[B32] McNeal F. H. Koznak C. F. Smith E. P. Tate W. S. Russell T. S. (1971). A uniform system for recording and processing cereal research data. *USDA-ARS Bull.* 42, 34–121.

[B33] Nasuda S. Friebe B. Gill B. S. (1998). Gametocidal genes induce chromosome breakage in the interphase prior to the first mitotic cell division of the male gametophyte in wheat. *Genetics* 149, 1115–1124. 9611219 10.1093/genetics/149.2.1115 PMC1460171

[B34] Ozkan H. Levy A. A. Feldman M. (2001). Allopolyploidy-induced rapid genome evolution in the wheat (*Aegilops – Triticum*) Group. *Plant Cell* 13 1735–1747. 10.1105/TPC.010082 11487689 PMC139130

[B35] Prażak R. Paczos-Grzęda E. (2013). Characterization of *Aegilops kotschyi* Boiss. x *Triticum aestivum* L. hybrid lines. *Acta Agrobot.* 66 109–120. 10.5586/aa.2013.057

[B36] Qi L. Friebe B. Zhang P. Gill B. S. (2007). Homoeologous recombination, chromosome engineering and crop improvement. *Chromosom. Res.* 15 3–19. 10.1007/s10577-006-1108-8 17295123

[B37] Rawat N. Tiwari V. K. Singh N. Randhawa G. S. Singh K. Chhuneja P. (2009). Evaluation and utilization of *Aegilops* and wild *Triticum* species for enhancing iron and zinc content in wheat. *Genet. Resour. Crop Evol.* 56 53–64. 10.1007/s10722-008-9344-8

[B38] Riley R. Chapman V. (1958). Genetic control of the cytologically diploid behaviour of hexaploid wheat. *Nature* 182 713–715. 10.1038/182713a0

[B39] Rodriguez-Algaba J. Sørensen C. K. Labouriau R. Justesen A. F. Hovmøller M. S. (2020). Susceptibility of winter wheat and triticale to yellow rust influenced by complex interactions between vernalisation, temperature, plant growth stage and pathogen race. *Agronomy* 10:13 10.3390/agronomy10010013

[B40] Roelfs A. P. (1988). An international system of nomenclature for *Puccinia graminis* f. sp. *tritici*. *Phytopathology* 78 526–533. 10.1094/phyto-78-526

[B41] Ruban A. S. Badaeva E. D. (2018). Evolution of the S-Genomes in Triticum-Aegilops alliance: evidences from chromosome analysis. *Front. Plant Sci.* 9:1756. 10.3389/fpls.2018.01756 30564254 PMC6288319

[B42] Schneider A. Molnár I. Molnár-Láng M. (2008). Utilisation of Aegilops (goatgrass) species to widen the genetic diversity of cultivated wheat. *Euphytica* 163 1–19. 10.1007/s10681-007-9624-y

[B43] Sears E. R. (1972). “Chromosome engineering in wheat,” in *Stadler Genet. Symp*, eds Kimber G. Redei G. P. (Columbia: University of Missouri), 23–38.

[B44] Sears E. R. (1977). An induced mutant with homoeologous pairing in wheat. *Can. J. Genet. Cytol.* 19 585–593. 10.1139/g77-063

[B45] Singh B. D. Singh A. K. (2015). *Marker-assisted plant breeding: principles and practices.* India: Springer 10.1007/978-81-322-2316-0

[B46] Smit C. (2013). *Pyramiding of novel rust resistance genes in wheat, utilizing marker assisted selection and doubled haploid technology.* Master of Science Thesis, Stellenbosch: Stellenbosch University, 1–123.

[B47] Tsujimoto H. Tsunewaki K. (1985). Gametocidal genes in wheat and its relatives. II. Suppressor of the chromosome 3C gametocidal gene of *Aegilops triuncialis*. *Can. J. Genet. Cytol*. 27, 178–185. 10.1139/g85-027

[B48] Veatch-Blohm M. E. (2007). Principles of plant genetics and breeding. *Crop Sci.* 47:1763 10.2135/cropsci2007.05.0002br

[B49] Wojciechowska B. Pudelska H. (2002). Production and morphology of the hybrids *Aegilops kotschyi × Secale cereale* and *Ae. biuncialis × S. cereale*. *Genet. Plant Acad.* 43 279–285. 12177517

[B50] Wulff B. B. H. Moscou M. J. (2014). Strategies for transferring resistance into wheat: from wide crosses to GM cassettes. *Front. Plant Sci.* 5:692. 10.3389/fpls.2014.00692 25538723 PMC4255625

[B51] Zohary D. (1999). Monophyletic vs. polyphyletic origin of the crops on which agriculture was founded in the Near East. *Genet. Resour. Crop Evol.* 46 133–142. 10.1023/A:1008692912820

[B52] Zohary D. Feldman M. (1962). Hybridization between amphidiploids and the evolution of polyploids in the wheat (*Aegilops-Triticum*) group. *Evolution* 16 44–61. 10.2307/2406265

