# Supplementary material for: Development and Cytomolecular Identification of Monosomic Alien Addition and Substitution Lines of Triticale (×Triticosecale Wittmack) With 2Sk Chromosome Conferring Leaf Rust Resistance Derived From Aegilops kotschyi Boiss
Source: Front Plant Sci. 2020 Dec 14;11:509481. doi: 10.3389/fpls.2020.509481 (PMC7767841; doi:10.3389/fpls.2020.509481)
Supplement: Supplementary file 1 [file Table_1.docx]

Supplementary table 1. Analysis of variance (ANOVA) and Tukey’s HSD test for leaf rust infection scores (collected in three timepoints) for: 1) 1^st^ experiment for monosomic 2S^k^(2R) substitution plants; 2) 2^nd^ experiment for monosomic 2S^k^(2R) substitution plants; 3) 1^st^ experiment for triticale cv. Sekundo controls and 4) 2^nd^ experiment for triticale cv. Sekundo controls. HSD - the absolute (unsigned) difference between any two sample means required for significance at the designated level HSD[.05] for the .05 level; HSD[.01] for the .01 level.

| ***Data Summary*** | | | | | | |
| --- | --- | --- | --- | --- | --- | --- |
|  | Groups | | | | Total | |
|  | 1 | 2 | 3 | 4 |  |  |
| Number of scores | 120 | 120 | 120 | 120 | 480 | |
| Σ | 356 | 355 | 768 | 771 | 2250 | |
| Mean | 2.9667 | 2.9583 | 6.4 | 6.425 | 4.6875 | |
| ΣX^2^ | 1084 | 1071 | 4978 | 5021 | 12154 | |
| Variance | 0.2342 | 0.1747 | 0.5277 | 0.5658 | 3.3552 | |
| Std. Dev. | 0.4839 | 0.418 | 0.7265 | 0.7522 | 1.8317 | |
| Std. Error | 0.0442 | 0.0382 | 0.0663 | 0.0687 | 0.0836 | |
| ***ANOVA summary*** | | | | | | |
| Source | SS | df | MS | F | | P |
| Treatment  (between groups) | 1428.3417 | 3 | 471.1139 | 1267.61 | | <.0001 |
| Error | 178.7833 | 476 | 0.3765 |  |  | |
| Total | 1607.125 | 479 |  |  |  | |
| ***Tukey HSD test:* HSD_0.05_ = 0.2; HSD_0.01_ = 0.25** | | | |  |  |  |
| Mean of group 1 vs mean of group 2 | | | nonsignificant |  |  |  |
| Mean of group 1 vs mean of group 3 | | | P<.01 |  |  |  |
| Mean of group 1 vs mean of group 4 | | | P<.01 |  |  |  |
| Mean of group 2 vs mean of group 3 | | | P<.01 |  |  |  |
| Mean of group 2 vs mean of group 4 | | | P<.01 |  |  |  |
| Mean of group 3 vs mean of group 4 | | | nonsignificant |  |  |  |
