# Supplementary material for: Development and Cytomolecular Identification of Monosomic Alien Addition and Substitution Lines of Triticale (×Triticosecale Wittmack) With 2Sk Chromosome Conferring Leaf Rust Resistance Derived From Aegilops kotschyi Boiss
Source: Front Plant Sci. 2020 Dec 14;11:509481. doi: 10.3389/fpls.2020.509481 (PMC7767841; doi:10.3389/fpls.2020.509481)
Supplement: Supplementary file 2 [file Table_2.docx]

Supplementary table 2. Analysis of variance (ANOVA) and Tukey’s HSD test for stripe rust infection scores (collected in three timepoints) for: 1) 1^st^ experiment for monosomic 2S^k^(2R) substitution plants; 2) 2^nd^ experiment for monosomic 2S^k^(2R) substitution plants; 3) 1^st^ experiment for triticale cv. Sekundo controls and 4) 2^nd^ experiment for triticale cv. Sekundo controls.

| ***Data Summary*** | | | | | | |
| --- | --- | --- | --- | --- | --- | --- |
|  | Groups | | | | Total | |
|  | 1 | 2 | 3 | 4 |  |  |
| Number of scores | 120 | 120 | 120 | 120 | 480 | |
| Σ | 395 | 385 | 389 | 399 | 1568 | |
| Mean | 3.2917 | 3.2083 | 3.2417 | 3.325 | 3.2667 | |
| ΣX^2^ | 1371 | 1309 | 1335 | 1413 | 5428 | |
| Variance | 0.5949 | 0.6201 | 0.6218 | 0.7254 | 0.6386 | |
| Std. Dev. | 0.7713 | 0.7875 | 0.7885 | 0.8517 | 0.7991 | |
| Std. Error | 0.0704 | 0.0719 | 0.072 | 0.0778 | 0.0365 | |
| ***ANOVA summary*** | | | | | | |
| Source | SS | df | MS | F | | P |
| Treatment  (between groups) | 0.9667 | 3 | 0.3222 | 0.5 | | 0.682452 |
| Error | 304.9 | 476 | 0.6405 |  |  | |
| Total | 305.8667 | 479 |  |  |  | |
